# External Magnetic Field-Assisted Porosity Reduction in Plasma Arc-Based Wire Arc Additive Manufacturing

**DOI:** 10.3390/ma19163477

**Published:** 2026-08-17

**Authors:** Shinichi Tashiro, Dang Khoi Le, Huy Le Phan, Quang Huy Duong, Kieu A. Duong Nguyen, Toshifumi Yuji, Bin Xu, Manabu Tanaka

**Affiliations:** 1 Joining and Welding Research Institute, The University of Osaka, Osaka 567-0047, Japan; 2 Joining and Welding Research Institute HUST-UOsaka, Hanoi University of Science and Technology, Hanoi 11618, Vietnam; 3 Faculty of Education, Miyazaki University, Miyazaki 889-2192, Japan; 4 College of Mechanical & Energy Engineering, Beijing University of Technology, Beijing 100124, China

**Keywords:** wire arc additive manufacturing, plasma arc, porosity, external magnetic field

## Abstract

This study proposed porosity reduction technology for Wire Arc Additive Manufacturing (WAAM) of aluminum alloys that employ a static transversal External Magnetic Field (EMF) to control cathode spot (CS) behavior for the first time, thereby facilitating bubble release, mainly by accelerating the removal of oxide layer. This paper reported effects of EMF strength (Magnetic Flux Density: MFD) on porosity reduction. The pore diameter distributions showed that the number of pores at MFDs of 2 mT and 3 mT decreased to approximately half of that at 0 mT and 1 mT for pore diameters less than 60 μm, indicating that applying EMF with MFD higher than 2 mT especially contributed to reducing the pore formation. The observation presented that the Electrode Negative (EN) arc effectively melted the wire, and the Electrode Positive (EP) arc cleaned the oxide layer on the rear weld pool surface by CSs with EMF. There were two types of bubble release behavior: a weak eruption outside the arc root (Type A), and a strong eruption inside the arc root (or CS) (Type B). Type B is considered to be more effective for porosity reduction, being caused by oxide removal by CS. One possible explanation for this is that the gas bubbles trapped beneath the oxide layer could be strongly drawn out through CS.

## 1. Introduction

Additive Manufacturing (AM) is a process that fabricates a three-dimensional and seamless product by layering materials at a time [1]. This is suitable for fabrication of complex-shaped structures and has many advantages, such as significantly reducing materials and labor costs [1], making it one of the manufacturing technologies that has attracted particular attention in recent years. Machine learning-assisted in situ adaptive strategies for the control of defects and anomalies [2], and field-assisted technologies [3] for AM were reviewed in detail. AM that uses an arc as a heat source and wire as the material is called Wire Arc AM (WAAM) [4], and has the remarkable advantage of being applicable to products larger than 10 m [5]. Microstructures of WAAM component using Inconel 718 [6], 304 Steel [7] and Ti6Al4V/Inconel 718 composites [8] were investigated.

**Figure 1:**
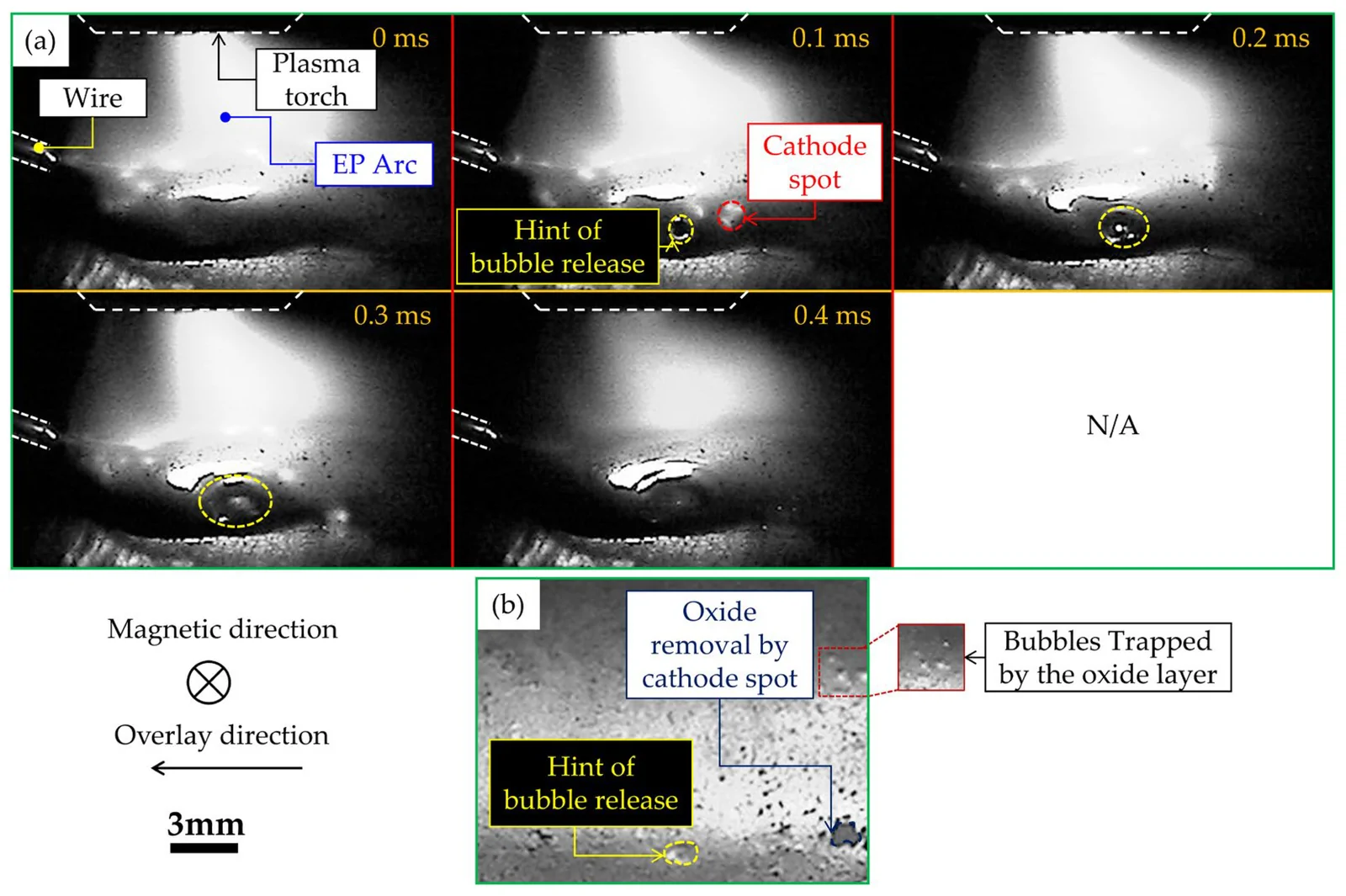
Examples for (**a**) the bubble release in the weld pool, cathode spot during electrode positive duration, and (**b**) the trapped bubbles by oxide layer, the oxide removal by the cathode spot, and the mark of a released bubble on the overlayer’s surface.

WAAM is applicable to various types of metallic materials, but the amount of porosity generated increases significantly, especially when using aluminum alloys [9,10]. When atmospheric gas is absorbed into the weld pool in large quantities, or when low-boiling point alloying elements contained in the weld pool vaporize, bubbles are generated. The bubbles are released from the weld pool by buoyancy or flow phenomena as shown in Figure 1. Hydrogen is considered to have a particularly significant influence as a gas species that causes this bubble generation because of large difference in solubility between the liquid and solid phases [11]. Water vapor from the surrounding air entrained in the shielding gas, moisture adhering to the surface of the metal wire (filler material), and that on the surface of the previously deposited metal layer are considered the primary sources of hydrogen gas [9]. If these bubbles are not released and the metal is solidified, small holes called pores are formed within the metal after solidification. These pores cause decrease in mechanical properties and stress concentration leading to the starting point for cracks.

In-process technologies for reducing porosity including the use of laser-arc hybrid welding [12,13] and the application of ultrasonic waves [14,15] have been developed. While these methods aim to reduce porosity mainly by promoting the bubble release, achieved through the stirring of weld pool or the expansion of the molten region, those face certain drawbacks: laser-arc hybrid welding requires expensive and large-scale equipment, and ultrasonic application methods impose significant limitations on the size and shape of the workpiece, as the ultrasonic waves must be applied to the substrate.

In addition to the above technologies, External Magnetic Field (EMF) can also be applied to WAAM. There are various methods for applying EMF; through interaction with the arc current, it generates a strong Lorentz force capable of altering the flow fields of the arc and the weld pool [16]. Corradi et al. generated EMF by installing two coils at a 90-degree angle on the sides of a welding torch and supplying alternating currents of different phases with them [17]. This approach was shown to be effective for fabricating thinner walls by minimizing weld pool overflow and, under certain conditions, improving deposition efficiency. Wang et al. investigated the effects of applying a longitudinal static EMF on the microstructure and mechanical properties of Inconel 625 alloy [18]. The results demonstrated that the application of EMF refined the dendritic structure, suppressed segregation, and effectively improved the mechanical properties of the Inconel 625 alloy. Zhao et al. applied an axial EMF using alternating current and conducted a comprehensive and comparative investigation into the effects of the EMF on arc behavior, metal transfer, deposit geometry, microstructure, and mechanical properties [19]. The results revealed that increasing excitation current promotes arc plasma expansion, thereby reducing heat flux density and increasing bead width while decreasing bead height and penetration depth. Increasing the excitation frequency was found to produce the opposite effects. Wang et al. applied a uniquely designed External Compound Magnetic Field (ECMF) system to simultaneously improve the efficiency and forming accuracy of Directed Energy Deposition (DED) [20]. This process installed three coils around the welding torch to dynamically control EMF by supplying pulsed currents with different phases to each coil. This novel method achieved high deposition rates in the DED process while maintaining excellent formability and mechanical properties. Furthermore, it was demonstrated that the application of ECMF facilitates the bubble release through the stirring and spreading of the weld pool for single layer deposition. As the aforementioned research findings [16,17,18,19,20] indicate, the behavior of the arc and weld pool varies significantly depending on the method of EMF application. In many cases, the objective of applying EMF is to improve mechanical properties or control the shape of the WAAM component. The paper [20] demonstrates that applying EMF to a single layer reduces porosity through stirring and expansion of the weld pool; however, the results of applying this technique to multiple layers have not been evaluated.

Yi et al. suggested that the formation of an oxide layer on the weld pool surface behind the welding torch hinders bubble release, thereby increasing the likelihood of pore formation [9]. Although details of this mechanism are not yet clarified due to difficulty of direct observation, the suggested mechanism is considered reasonable according to the following reasons: 1. Yuan et al. observed by synchrotron X-ray imaging that subsurface vapor bubbles in electron beam melting remained beneath the free surface before bursting explosively, indicating that bubbles are not always released immediately after reaching the weld pool surface [21]. 2. Although the exact reoxidation time under process conditions has not yet been directly quantified, previous studies suggest that freshly exposed aluminum surfaces can begin to oxidize on a millisecond timescale, and that molten aluminum can rapidly develop a continuous oxide layer ranging from a few nanometers to approximately 100 nm in thickness, depending on oxygen availability, temperature, and exposure time [22,23,24]. 3. Since this thin oxide layer possesses a strength exceeding 100 MPa, it is thought to prevent the release of bubbles [22].

When a Variable-Polarity Plasma Arc (VPPA) [25,26] is used as the WAAM heat source, the oxide layer on the aluminum surface is removed by the cleaning action of the Cathode Spot (CS) generated on the weld pool during the Electrode Positive (EP) phase [27,28,29]. However, the weld pool in the trailing region undergoes reoxidation because the surface protection provided by the shielding gas is less effective there. When applying a static transversal EMF, CSs can be controlled to shift along the weld line toward the trailing weld pool. In this case, CSs are expected to strongly assist the oxide removal, facilitating bubble release to reduce porosity. To the best of the author’s knowledge, no research has been found that attempts this approach to reduce porosity in WAAM.

This study proposes a porosity reduction technology that employs static transversal EMF to control CS behavior, thereby facilitating bubble release, mainly by accelerating the removal of oxide layer, being a new approach distinct from the above conventional methods to stir and expand the weld pool. This EMF application method is consistent with the methodology of a previous study on aluminum welding [30]. Key advantages of this technology include its feasibility via inexpensive compact auxiliary equipment and its minimal constraints regarding the size or shape of the deposited component. Hereafter, EMF refers to the static transversal EMF for simplicity.

In this study, VPPA and a ER5356 wire were utilized as the WAAM heat source and feedstock material, applying an EMF strength (Magnetic Flux Density: MFD) of 0 mT, 1 mT, 2 mT and 3 mT. In particular, effects of MFD on porosity diameter distribution and Porosity Area Percentage (PAP) were discussed for realizing porosity reduction in this paper. The porosity reduction mechanism using this approach was also suggested through observation results of CS, arc and weld pool behaviors with a laser-assisted High-Speed Video Camera (HSVC).

## 2. Experimental Methods

### 2.1. Wire Arc Additive Manufacturing

**Figure 2:**
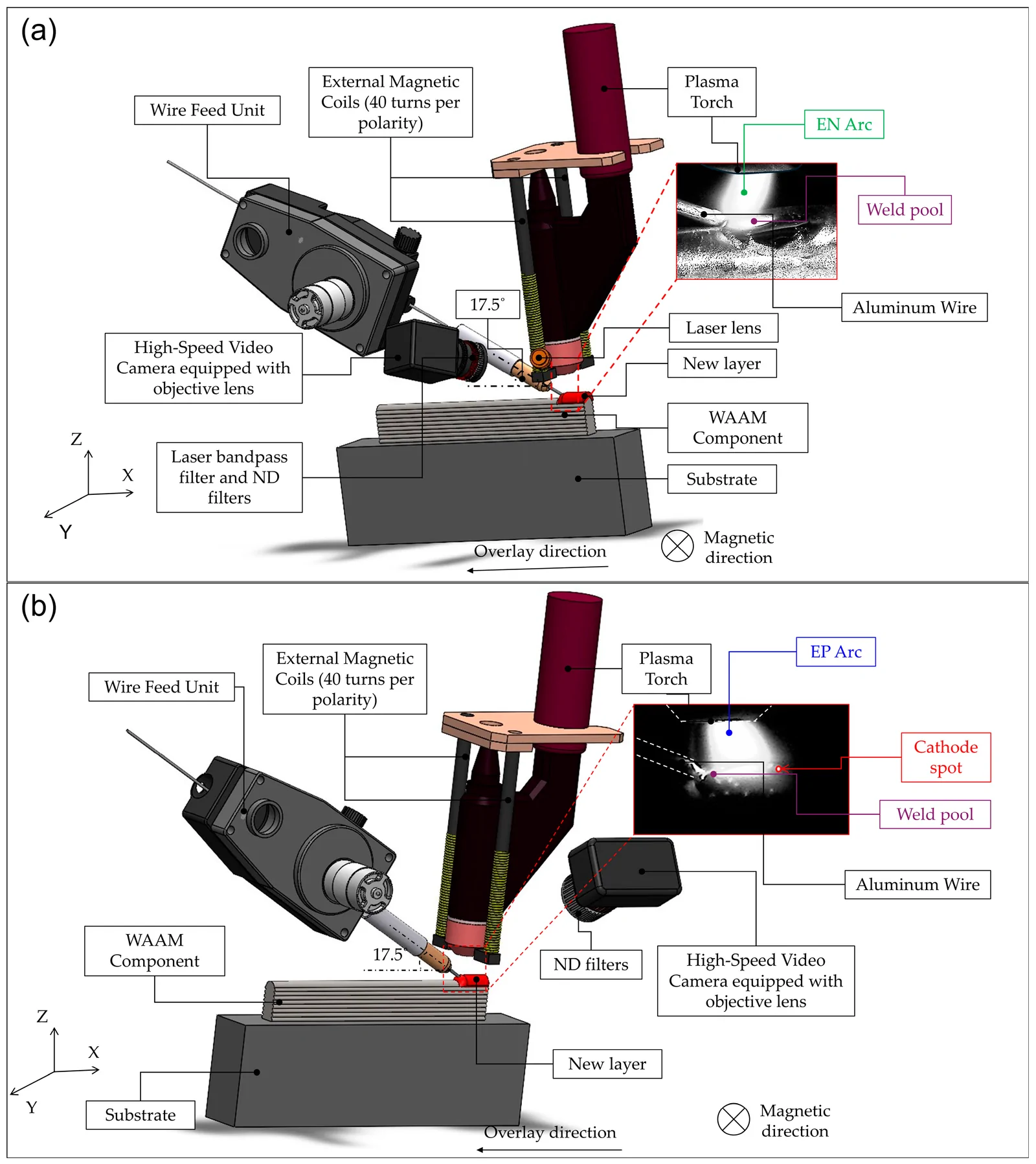
Schematics for experimental setups for (**a**) weld pool and arc appearance observations with the reflected laser-assisted system, and (**b**) cathode spot observation.

Figure 2 shows the schematics of experimental setup for the VPPA-based WAAM. The WAAM component was done on the thin-edge surface of A5052P aluminum (TP Giken, Ltd., Osaka, Japan) plate using a 1.2 mm diameter ER5356 aluminum wire (Kobe Steel, Ltd., Kanagawa, Japan) as a feedstock material. The chemical composition of the plate and feedstock material are presented in Table 1.

The system consisted of a VPPA power source (NW-300ASR-2, Nippon Steel Welding & Engineering Co., Ltd., Tokyo, Japan), a tailored wire feed unit, a customized EMF device, and a customized 3-axis moving system. The wire feed unit was set at an angle of 17.5° relative to the plate surface. The EMF device utilized two coils acting as two polarities, each consisting of 40 turns of 1 mm diameter copper wire.

All trials were conducted in the VP mode. The EP ratio was fixed at 30%, and a polarity-switching frequency was set to 50 Hz. The Wire Feed Speed (WFS) and the Magnetic Flux Density (MFD) were controlled by adjusting the voltage of the 24 V DC power supplies (SPS3010, Kungber, Shenzhen, China). WFS values were determined by averaging ten calculations at each voltage level applied. For each value, WFS was calculated by dividing the fed wire length by the corresponding feeding time. MFD was measured directly at 2 mm under the plasma torch nozzle by using gaussmeter (WT163, Weite Magnetic Technology Co., Ltd., Shangqiu, China) with an accuracy of 2%.

Four MFD levels, namely 0 mT, 1 mT, 2 mT, 3 mT, were applied to investigate the effect of EMF on the micro-porosity reduction in VPPA-based WAAM with aluminum wire. The 3-axis moving system was controlled by Arduino (UNO R3 ATmega328P CH340, China) commands generated in Candle software (Version 1.2.15b). For simplicity, this system is omitted from the schematic.

In VPPA, as presented in [25], the heat input during the Electrode Negative (EN) period is lower than that during the EP period, heat flux for the former is much higher due to the concentration of arc column. Therefore, the EN arc is used for melting the wire. In contrast, the EP current is proportional to the strength of oxide removal by CSs. Based on these characteristics, the EN and EP current levels were adjusted for each specific layer to meet the target of this study. The detailed parameters are presented in Table 2.

For all WAAM components, the first layer was deposited without applying EMF. During this layer, the EN current was set higher than the EP current. The substrate was initially at a room temperature (approximately 20 °C) and required sufficient heat input to ensure stable deposition of the continuous 80 mm bead. Notably, the torch remained stationary for approximately 5 s before the workpiece started moving. This procedure allowed the substrate temperature to become sufficiently uniform near the deposition region.

From the second layer onward, the EN current was set lower than the EP one. This strategy was adopted to take advantage of removing the oxide layer on the bead surface and facilitate bubble release [21,22,23]. However, the EN current remained sufficiently high to ensure stable wire melting. Therefore, the EN current was limited to only about 10% below the EP current. The EN and EP current levels were further adjusted at different deposition stages to avoid excessive heat accumulation as the wall height increased.

Current and voltage signals were recorded using a data logger (NR-500/NR-HV04, KEYENCE Corporation, Osaka, Japan) with an accuracy of ±0.05% and the sampling rate of 10 kHz. The welding current was measured using a clamp meter (3285, Hioki E.E. Corporation, Nagano, Japan) and transmitted to the data logger. The arc voltage was measured directly between the positive and negative welding cables. During pilot arc generation and arc ignition, a high voltage is applied for the main arc ignition. This voltage may exceed the safe operating range of the acquisition system and potentially damage the voltage measurement channel. Therefore, the Bayonet Neill-Concelman (BNC) connector for voltage measurement was connected only after a stable main arc had been established and the pilot arc had been extinguished. The couples of these waveforms will be presented later.

Argon gas was used as both shielding and plasma gases. The plasma gas flow rate of 1 L min^−1^ was used to maintain sufficient penetration and a stable transport channel, while the shielding gas flow rate of 25 L min^−1^ was employed to prevent atmospheric contamination. The arc length was consistently maintained at around 5 ± 1 mm throughout all trials. The travel speed of 3 mm s^−1^ for all layers, while the WFS was adjusted based on the current levels applied to ensure stable wire melting and deposition. The experiment was conducted at an air temperature of 20 °C and a relative humidity of 55%.

### 2.2. High-Speed Camera Observations

The simultaneous observation of the weld pool and arc appearance is illustrated in Figure 2a. HSVC (Memrecam Q1v, Nac Image Technology, Tokyo, Japan) coupled with a 647 nm laser illumination system (Cavilux HF, Cavitar, Ltd., Tampere, Finland) was employed. The camera was set on the side, on the front side of the weld pool at an angle of 5° to the substrate surface, while the laser was positioned to the right of the camera, on the back side of the weld pool at the same angle to provide reflected light. The angle between the camera and laser optical axes was 45°.

An objective lens (Micro-NIKKOR, Nikon Corporation, Tokyo, Japan) featuring a 200 mm focal length and a maximum aperture ratio of 1:4 was attached to the camera to obtain sufficient magnification of the weld pool. The high-speed images were recorded at a resolution of 640 × 288 pixels, a frame rate of 10,000 fps, and an exposure time of 6 μs. The objective lens aperture was set to f/16. The pulsed laser illumination with the wavelength of 647 nm was synchronized with the camera at 10,000 Hz. The bandpass filter, supplied with the laser system, with the central wavelength of 647 nm and the full width at half maximum of 20 nm was mounted on the objective lens to reduce the arc brightness and radiation. This setup also captured CS behavior. However, the combination of reflected laser illumination and intense arc radiation reduced the clarity of the CS images. Therefore, an additional observation method was employed to obtain clearer CS behavior and to better investigate its potential role in promoting bubble release.

Figure 2b illustrates the experimental setup for the CS observation. In this setup, only the HSVC Q1v and the same Micro-NIKKOR lens used in the previous observation were employed. The high-speed images were recorded at the same resolution and frame rate as those in the previous observation. The exposure time was set to 6 μs, and the objective lens aperture was set to f/8. To suppress arc brightness and radiation, three Neutral Density (ND) filters were mounted on the camera lens. The filter combination consisted of two ND-8 filters and one ND-2 filter. Notably, both observation methods used identical image signal processing settings, including high gain, normal gamma, and high to ensure consistency.

## 3. Results and Discussion

### 3.1. Analysis of the Wall Structure

**Figure 3:**
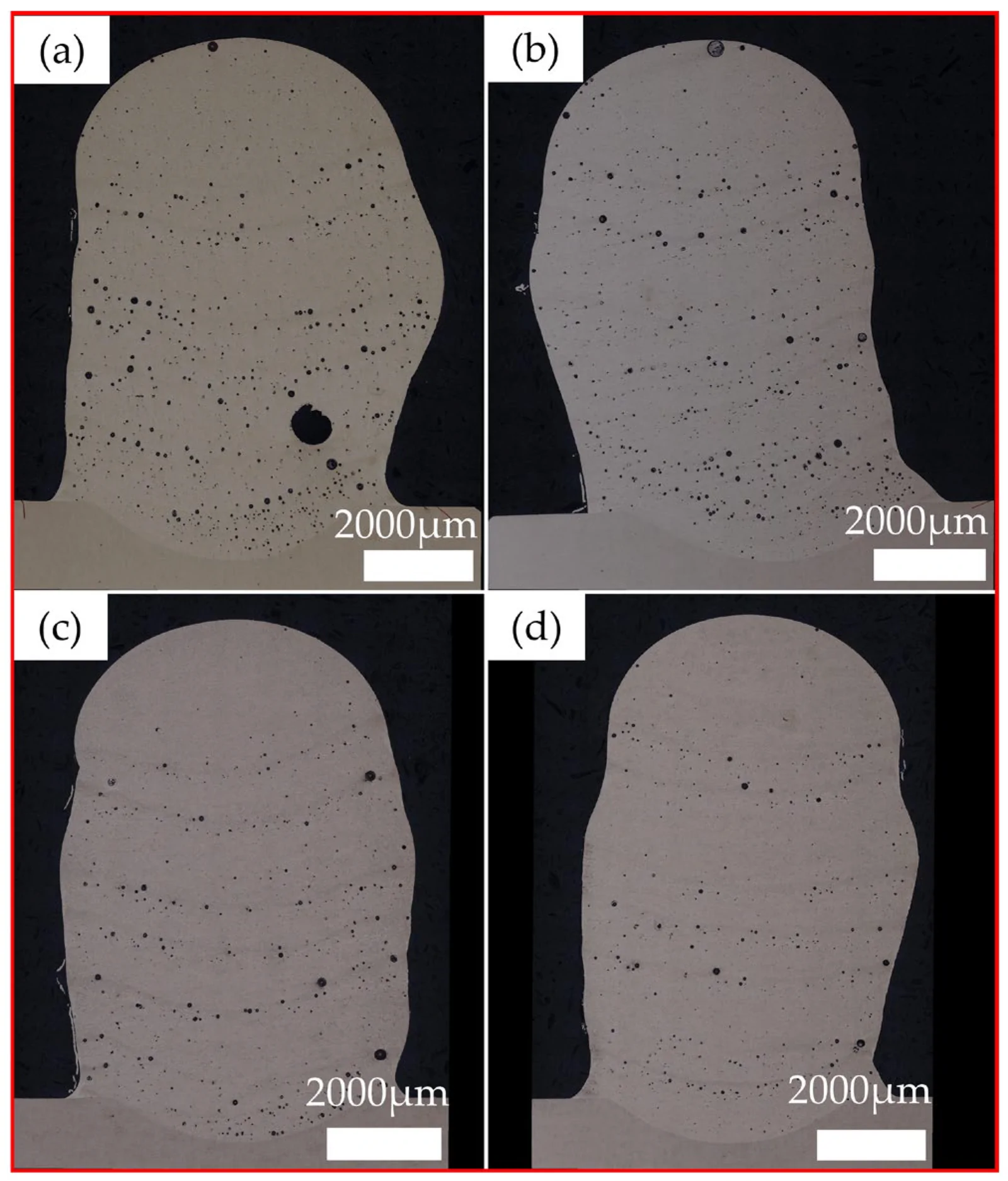
Cross sections of the wire arc additively manufactured components (**a**) at 0 mT, (**b**) 1 mT, (**c**) 2 mT, and (**d**) 3 mT.

Figure 3 shows the cross sections of the WAAM components at different MFDs: (a) 0 mT, (b) 1 mT, (c) 2 mT, and (d) 3 mT. All WAAM components showed comparable macroscopic appearances under the applied MFDs. Although slight differences in sidewall morphology could be observed, the wall height and width remained nearly consistence at 8.80 ± 0.04 mm, and 6.32 ± 0.04 mm, respectively, regardless of the applied MFDs. The values of the wall height and width were the averages of the three random cross-sectional area measurements for each WAAM component, where its structure was stable. The minor variations in sidewall morphology may be attributed to small fluctuations in the wire insertion position relative to the weld pool during deposition. Since similar features were observed under all conditions, the influence of the applied MFDs on the overall appearance appeared limited. This figure also indicates that most pores were concentrated in the interlayer regions between adjacent layers regardless of MFDs. Whereas, the top layer exhibited qualitatively few pores at MFDs of 2 mT and 3 mT.

To evaluate the pore distribution under the effects of the applied EMF, the cross-sectional specimens shown in Figure 3 were analyzed using a digital microscope (VHX-7000, KEYENCE Corporation, Osaka, Japan). The observations were conducted using a universal zoom lens (VH-Z100UR, KEYENCE Corporation, Osaka, Japan) with 200X magnification. The calibrated scale was added using the built-in function of the digital microscope. The results of the porosity analysis are presented below.

**Figure 4:**
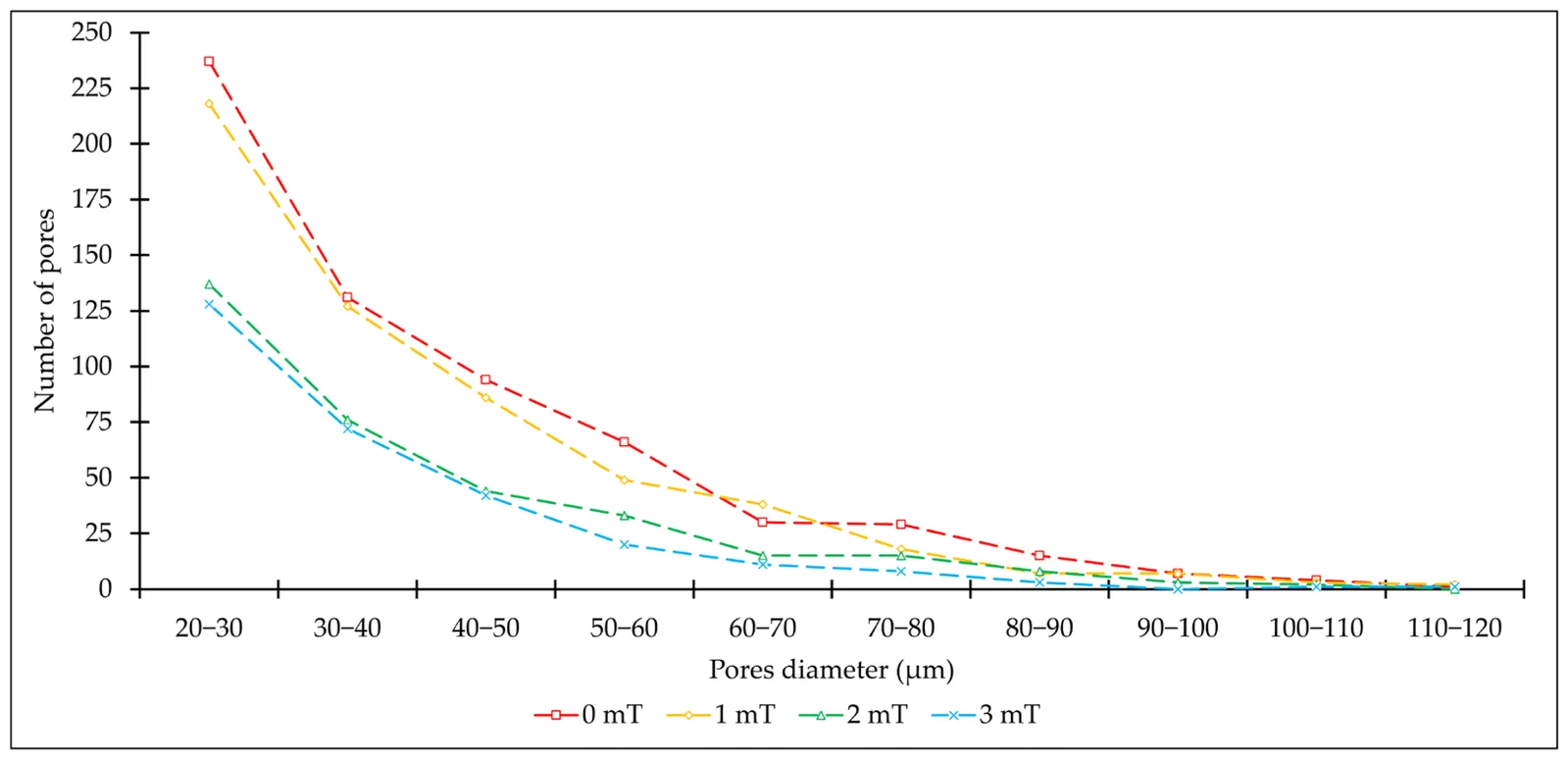
Pore diameter distributions at different magnetic flux densities.

Figure 4 shows the pore diameter distributions at different MFDs. The image captured by the VHX-7000 was opened in ImageJ software (Version 1.54g). The scale ratio used in VHX-7000 was referred to the “Set Scale” on ImageJ to get the conversion factor from pixels to μm. The image type was set to 8-bit image. Then, the Region of Interest (ROI)—only the WAAM component’s area—was manually selected and duplicated for the pore analysis. Because the grayscale contrast varied among the captured images, the brightness and contrast were adjusted individually before thresholding to improve the delineation of pore boundaries. The subsequent segmentation was verified by visual comparison with the original image. When necessary, the pore’s shapes were manually refined by restoring the missing portions of the pore boundaries. Subsequently, ImageJ’s thresholding function was applied to segment the pore regions from the ROI. The segmented image was then converted into a binary image and inverted so that the pores appeared as white objects against a black background for particle analysis. The pore’s area analysis was generated by using ImageJ’s “Analyze Particles” function. Although the analysis was restricted to the ROI, a small portion of the surrounding embedding resin remained in the segmented image after ROI selection. Because this region formed a single connected object that was substantially larger than any pore observed, an upper particle-size limit of 1,000,000 ${\mu m}^{2}$ was specified in “Analyze Particles” to exclude it from the pore measurements. Then, the data analyzed were displayed in ImageJ results table and exported to an Excel file for subsequent analysis. The exported data were used to calculate the equivalent diameter of each pore, to determine the pore distribution, and to generate the related charts. For diameter estimation, each pore was assumed to have an equivalent circular shape.

The results indicate that the number of pores monotonically decreased with increasing pore diameter, regardless of MFDs. The values were obtained from the analysis of the cross-sectional area enclosed by the boundary of each WAAM component, corresponding to approximately 54.4 mm^2^ ± 3.2 mm^2^.

Interestingly, the number of pores at MFDs of 0 mT and 1 mT was significantly higher than that at 2 mT and 3 mT for pore diameters ranging from 20 to <60 μm. Within this diameter range, the number of pores decreased from 237 to 66, from 218 to 49, from 137 to 33, and from 128 to 20 at MFDs of 0 mT, 1 mT, 2 mT, and 3 mT, respectively, as the pore diameter increased.

The difference in the number of pores became less pronounced for pore diameter ranging from 60 μm to <100 μm. For instance, in the pore diameter range of 80 μm–90 μm, the WAAM component at 0 mT contained 15 pores, whereas the corresponding values at 1 mT, 2 mT, 3 mT were 7, 8, and 3, respectively. The number of pores gradually reached zero for pore diameters above 100 μm regardless of MFDs. These results indicate that applying EMF with MFD higher than 2 mT especially contributed to reducing the pore formation in WAAM component.

For further evaluation of the effectiveness of porosity reduction under different MFDs, the Porosity Area Percentage (PAP) was calculated. Based on the pore areas measured using ImageJ and the cross-sectional area enclosed by the boundary of each WAAM component, the PAP was calculated as the ratio of the total pore area to the total cross-sectional area of the WAAM component.

**Figure 5:**
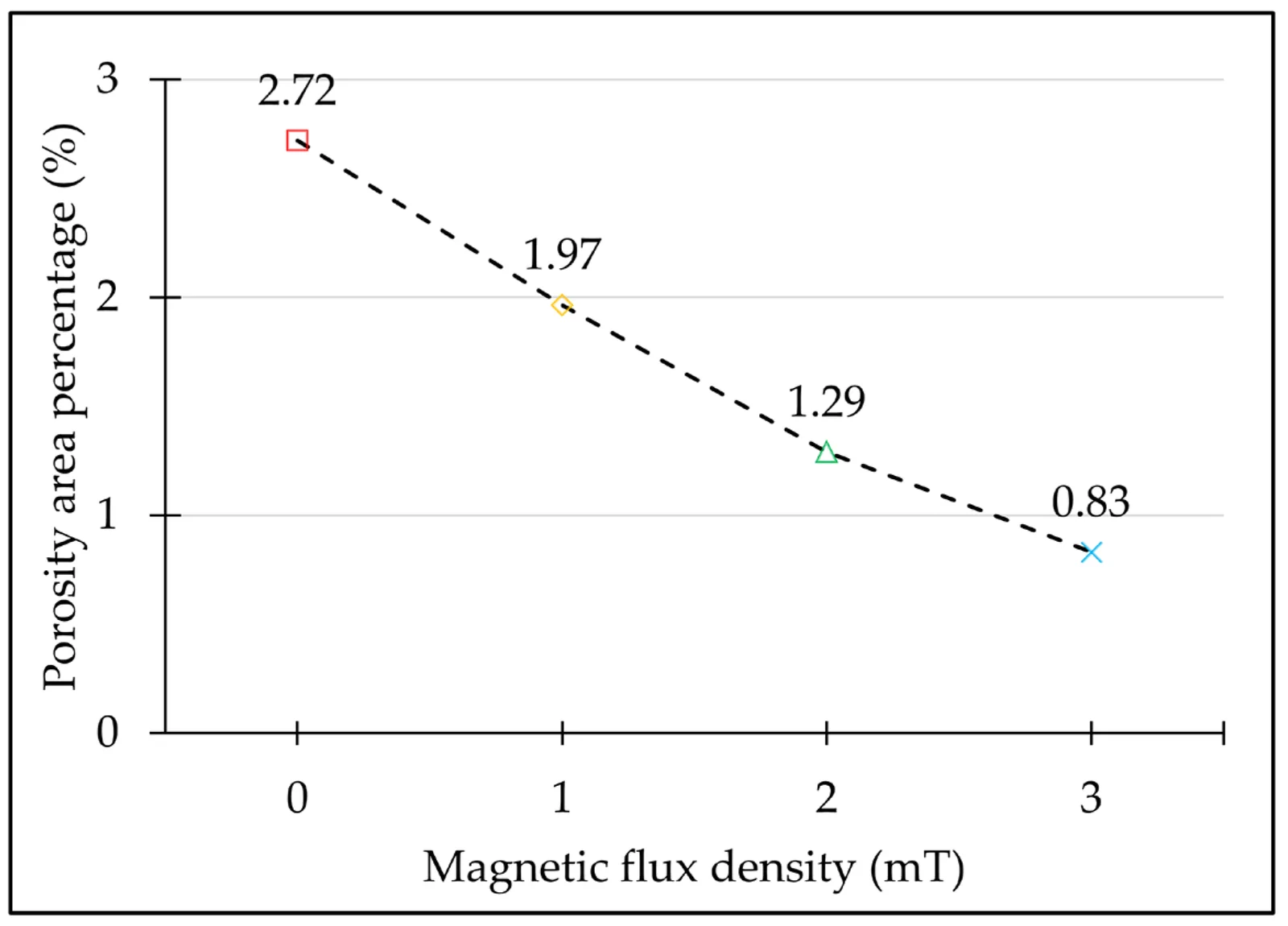
Porosity area percentage at different magnetic flux densities.

Figure 5 shows the porosity area percentage at different MFDs. The results indicate that increasing MFD reduced the area fraction occupied by porosity. As MFD increased from 0 mT to 3 mT, the PAP decreased from 2.72% to 0.83%, corresponding to an approximately 3.3-fold reduction.

### 3.2. Arc, Cathode Spots and Weld Pool Phenomena

This section presents the analysis of the weld pool, the arc appearance, and the cathode spot behavior based on high-speed observations. First, typical images of the weld pool and arc under both the EN and EP periods are presented to provide an overview of their characteristics at different MFDs. Next, time-sequential images captured during a current cycle are used to illustrate the weld pool behavior and arc appearance in greater detail. Finally, the typical images of the arc appearance under the EN and EP periods are presented for each applied MFDs. The detailed results are presented in the following parts.

**Figure 6:**
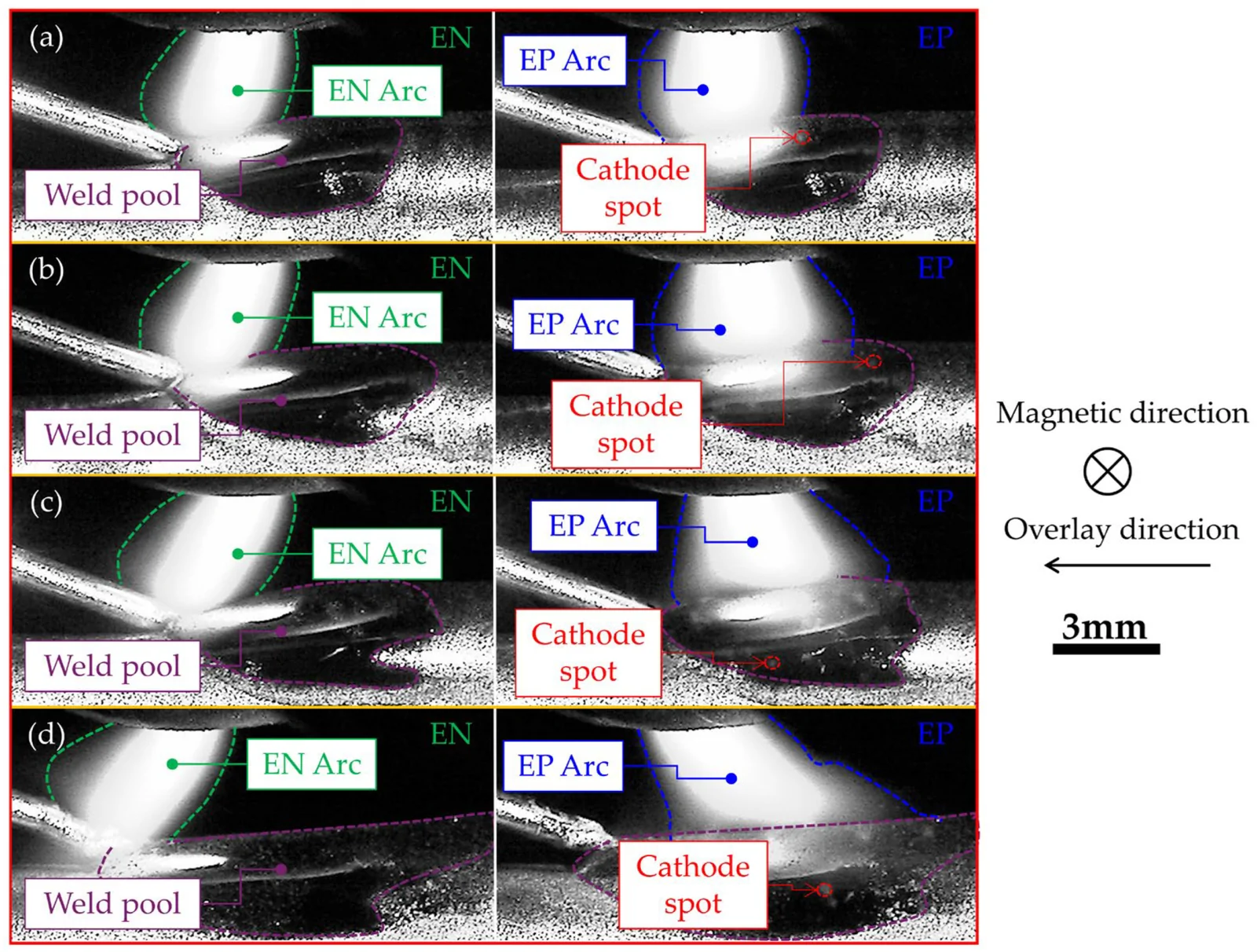
The typical images of the weld pool and arc appearances during the stable electrode negative (EN) and electrode positive (EP) periods at magnetic flux densities of (**a**) 0 mT, (**b**) 1 mT, (**c**) 2 mT, and (**d**) 3 mT.

Figure 6 shows the typical images of the weld pool behavior and the arc appearance during the stable EN and EP periods at MFDs of (a) 0 mT, (b) 1 mT, (c) 2 mT, and (d) 3 mT. In this figure, the EN arc, EP arc, weld pool, and CS are indicated by green, blue, violet, and red dashed lines, respectively. For consistency, the same labeling scheme is used throughout this paper.

The figure shows that increasing MFD broadened the weld pool boundary and enhanced arc deflection under both EN and EP conditions. This trend is particularly pronounced at 3 mT. The high-speed images also revealed that existence of CS. However, CS appeared blurred because the laser illumination and the high intensity of the arc radiation decreased its brightness. Thus, its behavior is discussed separately in a later section. As MFD increased, the EN arc root gradually shifted from the beginning of the weld pool to the un-melted feedstock material. In contrast, the EP arc root moved in the opposite direction toward the previously deposited region of the processing layer. The detailed evolution of the weld pool and arc behavior is presented in the following section.

**Figure 7:**
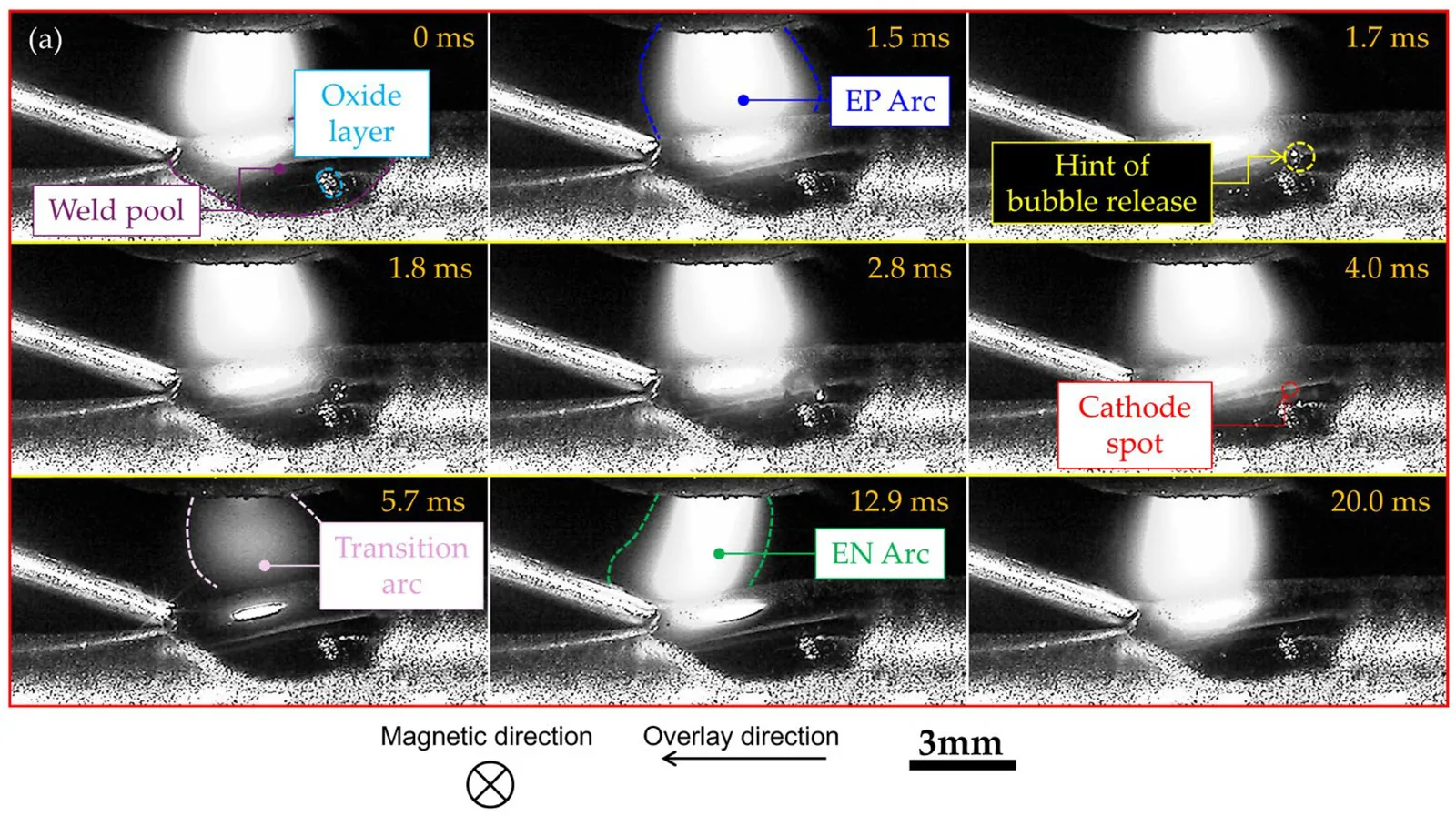

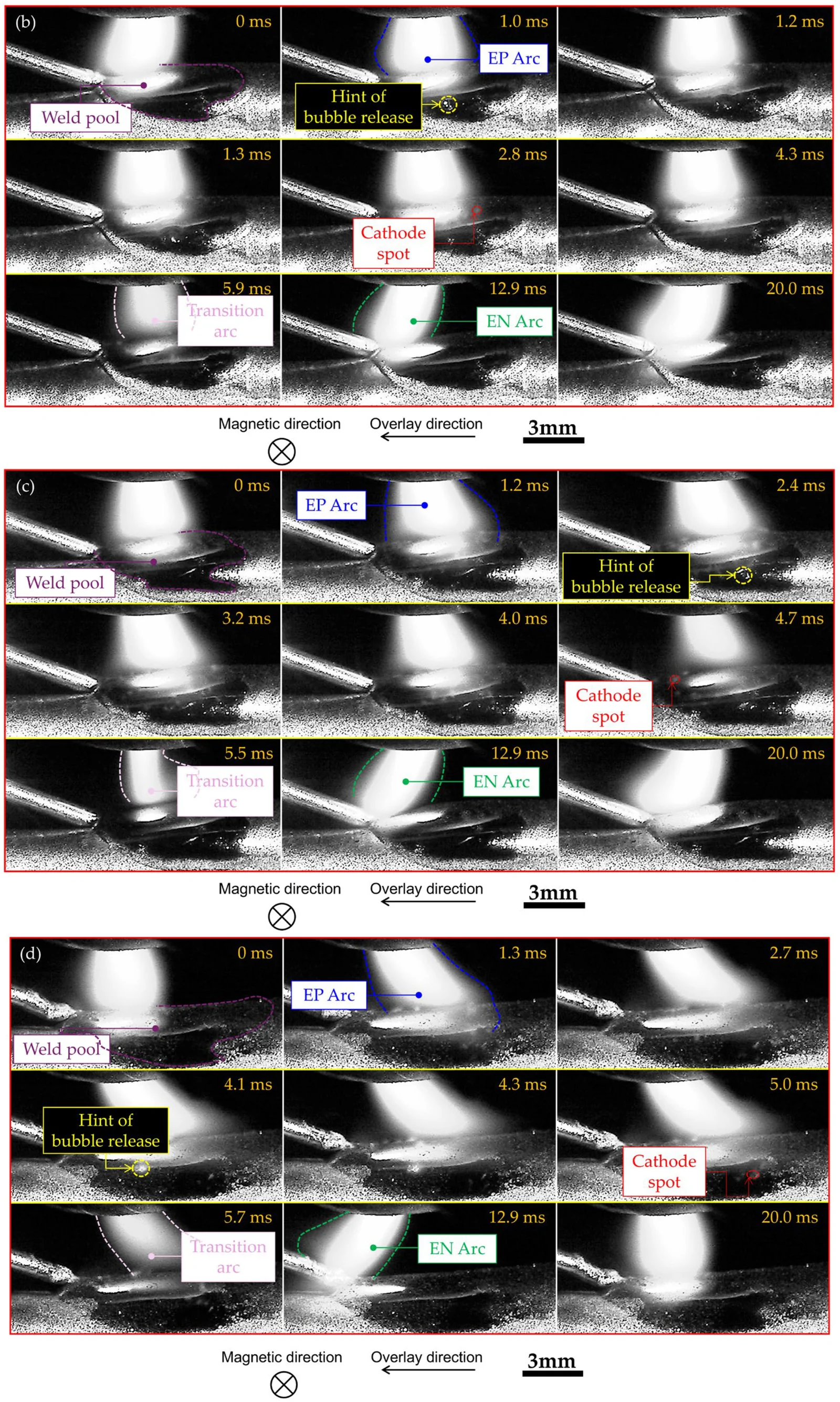
The time-sequential high-speed images of a current cycle recorded for the eighth layer including the weld pool behavior, the electrode negative (EN) and electrode positive (EP) arcs, the example of cathode spots under EP period, and the hint of bubble release for Type A at the magnetic flux densities of (**a**) 0 mT, (**b**) 1 mT, (**c**) 2 mT, and (**d**) 3 mT.

**Figure 8:**
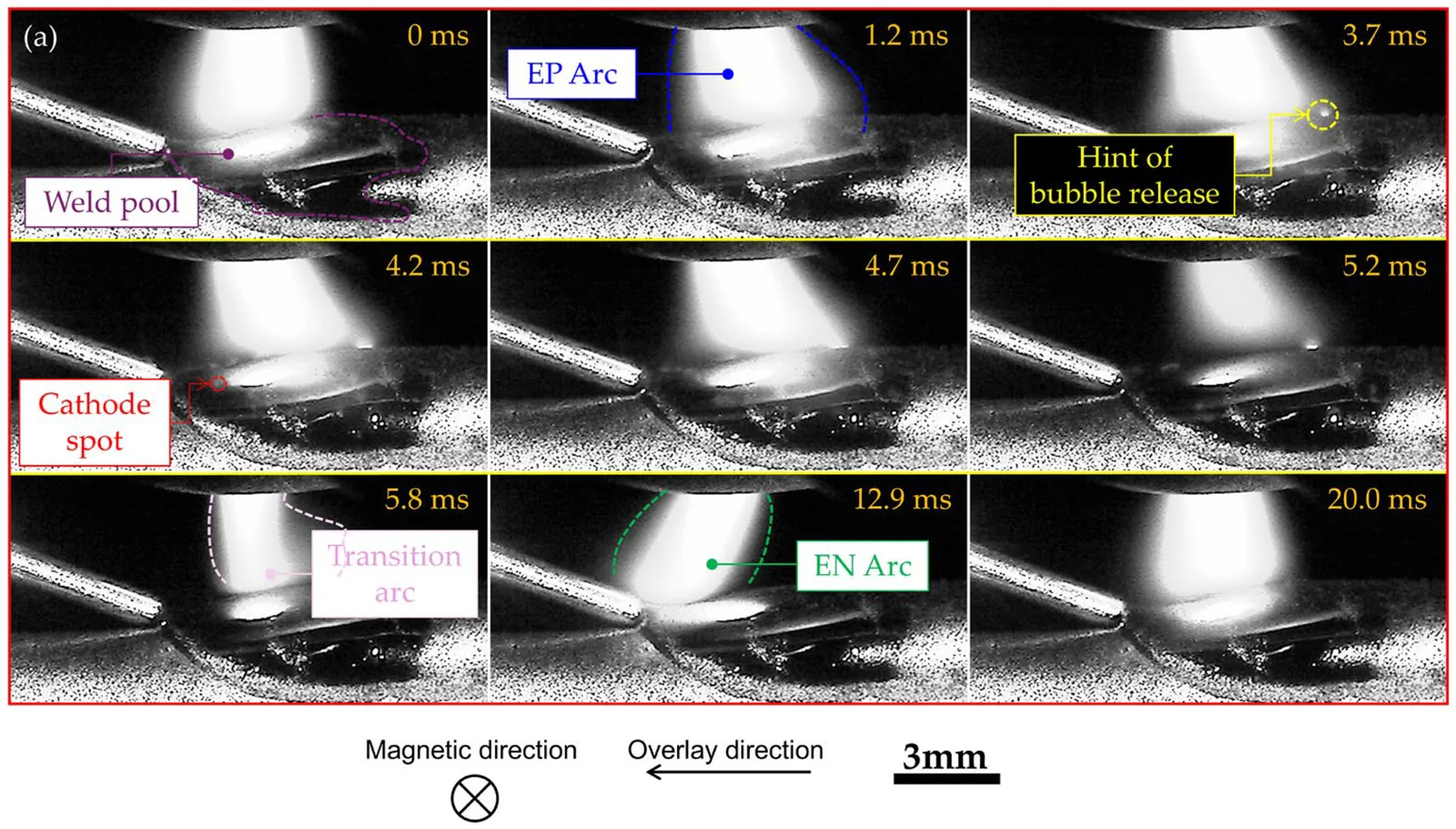

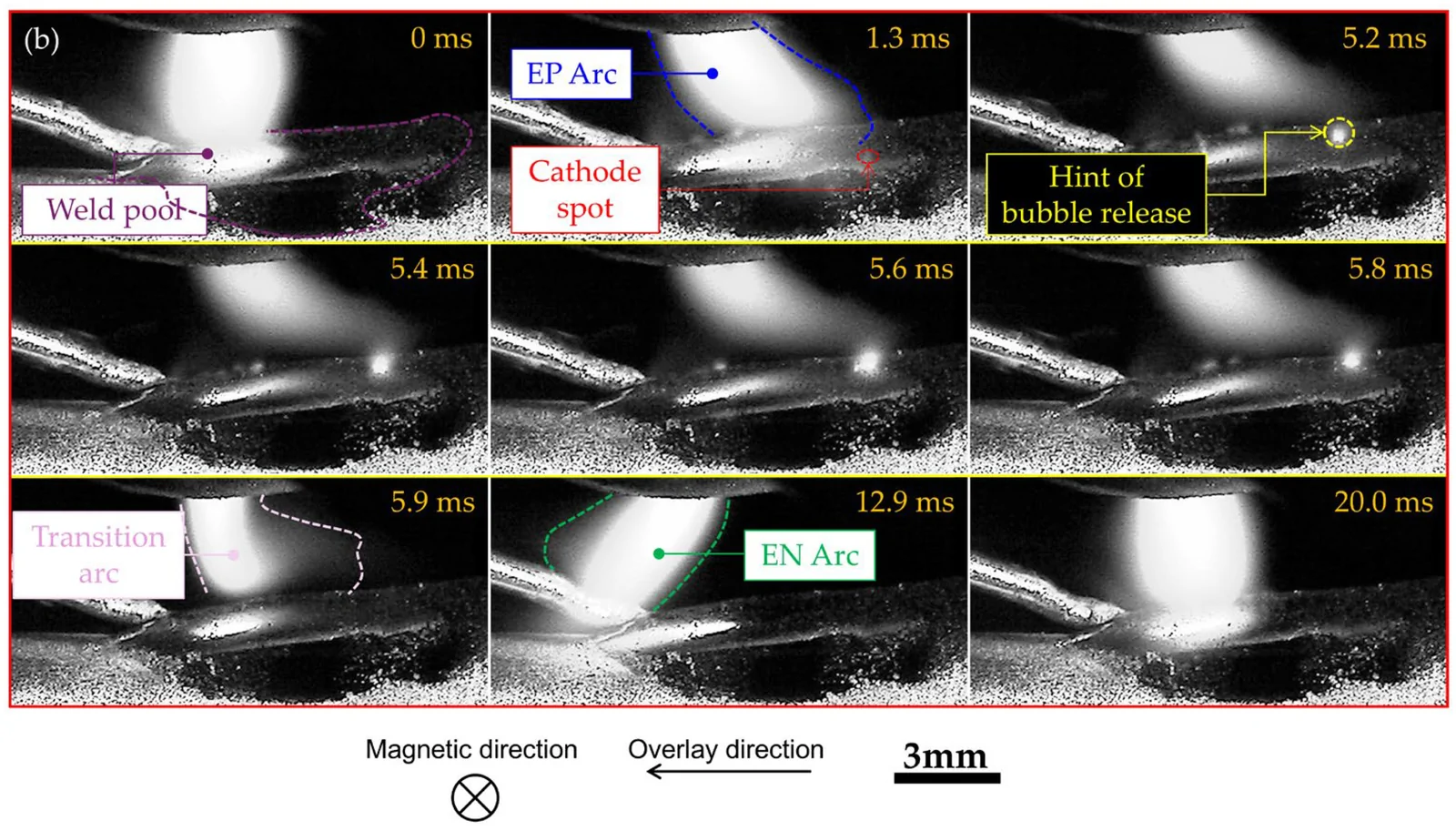
The time-sequential high-speed images of a current cycle recorded for the eighth layer including the weld pool behavior, the electrode negative (EN) and electrode positive (EP) arcs, the example of cathode spots under EP period, and the hint of bubble release for Type B at MFDs of (**a**) 2 mT, and (**b**) 3 mT.

Analysis of the high-speed images captured using reflected laser-assisted observation revealed two types of bubble release behavior: a weak eruption outside the arc root (Type A) as already presented in Figure 1, and a strong eruption inside the arc root with the appearance of a luminous spot (Type B). The detailed observations of these phenomena, together with the arc appearance, weld pool behavior, and CSs under EP period are presented in Figure 7 and Figure 8.

Figure 7 shows time-sequential high-speed images of a current cycle recorded for the eighth layer including the weld pool behavior, the EN and EP arcs, the example of CSs under EP period, and the hint of bubble release for Type A under the different applied MFDs. The hint of bubble release events is indicated by the yellow dashed lines. The sequence begins at the start of the EP period and ends at the end of the EN period.

Figure 7a shows the weld pool behavior and arc appearance at MFD of 0 mT. The weld pool boundary remained nearly unchanged throughout the current cycle. After switching from the EN period to the EP period, the EP arc root moved from near the beginning of the weld pool to its central region within approximately 1.5 ms. It then remained at this position until the next transition to the EN period. In contrast, the EN arc root maintained a nearly constant position near the beginning of the weld pool throughout the EN period (from ~12.9 ms to 20.0 ms).

Interestingly, a bubble release event was captured during the EP period. The event first appeared at 1.7 ms near the trailing side of the weld pool as a small surface eruption. The affected region expanded at 1.8 ms and gradually disappeared by approximately 2.8 ms.

Figure 7b shows the weld pool behavior and arc appearance at MFD of 1 mT. Compared with the 0 mT condition, the weld pool extended further toward the trailing side of the processing layer. This expansion may be attributed to EP arc deflection under the applied EMF, which promoted remelting in the deposited region.

After the transition from the EN period of the previous cycle to the EP period, the EP arc root gradually moved away from the beginning of the weld pool and reached its furthest position at approximately 2.8 ms (when the EP arc was stable). It then remained in this region until the next transition to the EN period. In contrast, the EN arc root was distributed from the beginning of the weld pool to the wire tip.

A bubble release event was captured at 1.0 ms and 1.2 ms. It occurred near the root of the weld pool on the trailing side of the processing layer.

Figure 7c shows the weld pool behavior and arc appearance at MFD of 2 mT. As the MFD increased to 2 mT, the weld pool extended toward the trailing side of the processing layer to similar extent with 1 mT. Arc appearance was also similar to that observed under 1 mT, with the furthest arc-root position during the EP period remaining at approximately the same distance from the beginning of the weld pool.

A bubble release event was also observed at this MFD level. A representative example is indicated at 2.4 ms.

Figure 7d shows the weld pool behavior and arc appearance at MFD of 3 mT. The weld pool expansion to backward increased much compared to that at 2 mT. At this MFD level, the weld pool range expanded even further compared with other MFD conditions and covered most of the observed areas of the processing layer.

The arc deflection was the most pronounced at 3 mT. During the EP period, the arc root shifted furthest toward the trailing side of the weld pool. In contrast, during the EN period, the arc root was located on the un-melted wire tip. A representative bubble release event was captured at 4.1 ms.

To provide further insight into the bubble release behavior, especially at high MFDs, Figure 8 presents the time-sequential high-speed images of a current cycle recorded for the eighth layer including the weld pool behavior, the EN and EP arcs, the example of CSs under EP period, and the hint of bubble release for Type B at MFDs of (a) at MFD of 2 mT, and (b) at MFD of 3 mT.

Overall, the arc appearance, weld pool appearance, and CS behavior under EP during the current cycle were similar to those illustrated in Figure 7. However, a notable difference was observed in the bubble release behavior. At 2 mT, from 3.7 ms to 5.2 ms, and at 3 mT, from 5.2 ms to 5.8 ms, a luminous spot appeared within the arc root region on the back of the weld pool and seemed to gradually expand in the vertical direction, which was thought to be caused by the occurrence of upward plasma jet in CS. One possible explanation is that this luminous spot might indicate the bubble release from the weld pool. The high-pressure hydrogen gas inside the bubble trapped under the oxide layer was released into CS due to the oxide removal effect by CS. The laser-assisted HSVC observation used the bandpass filter with the central wavelength of 647 nm and the full width at half maximum of 20 nm. Therefore, if CS included the dense hydrogen plasma inside, it would emit strong radiation at the wavelength of 656.28 nm, thereby increasing the brightness of CS in the image. Such behavior was hardly observed at lower MFDs of 0 mT and 1 mT.

From the above results, it is hypothesized that hydrogen gas bubbles trapped beneath the oxide layer are strongly drawn out through CS. This phenomenon classified as Type B, frequently observed at 2 mT and 3 mT, is believed to be linked to the significant reduction in porosity at MFD levels of 2 mT and above, as shown in Figure 4. Accordingly, Type B is considered to be more effective than Type A.

**Figure 9:**
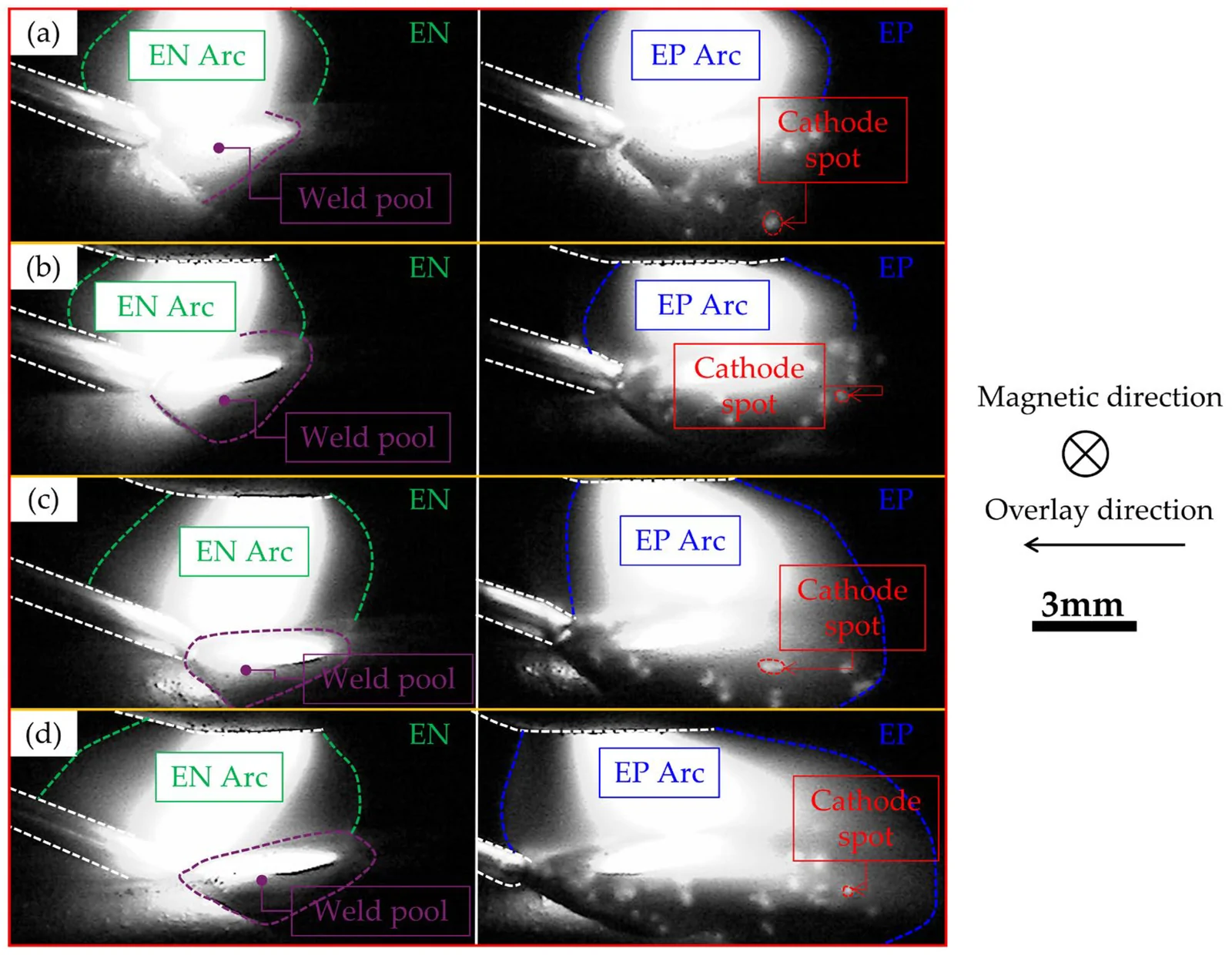
The typical images of arc appearance on the weld pool of the eighth layer at the magnetic flux densities of (**a**) 0 mT, (**b**) 1 mT, (**c**) 2 mT, and (**d**) 3 mT.

Figure 9 shows the typical images of arc appearance on the weld pool of the eighth layer at MFDs of (a) 0 mT, (b) 1 mT, (c) 2 mT, and (d) 3 mT. As MFD increased, both the EN and EP arcs became broader. Consistent with the observations in Figure 6, Figure 7 and Figure 8, the weld pool area also tended to expand.

The influence of EMF on CS behavior under EP period was more apparent in these images. Qualitatively, the number of visible CSs appeared to increase with increasing MFDs. In addition, the CS distribution became progressively widespread and covered a larger area of the weld pool surface. These observations suggest that the applied MFDs enhanced the CS activity in the trailing region of the weld pool covered by the oxide layer.

### 3.3. Current and Voltage Waveforms

**Figure 10:**
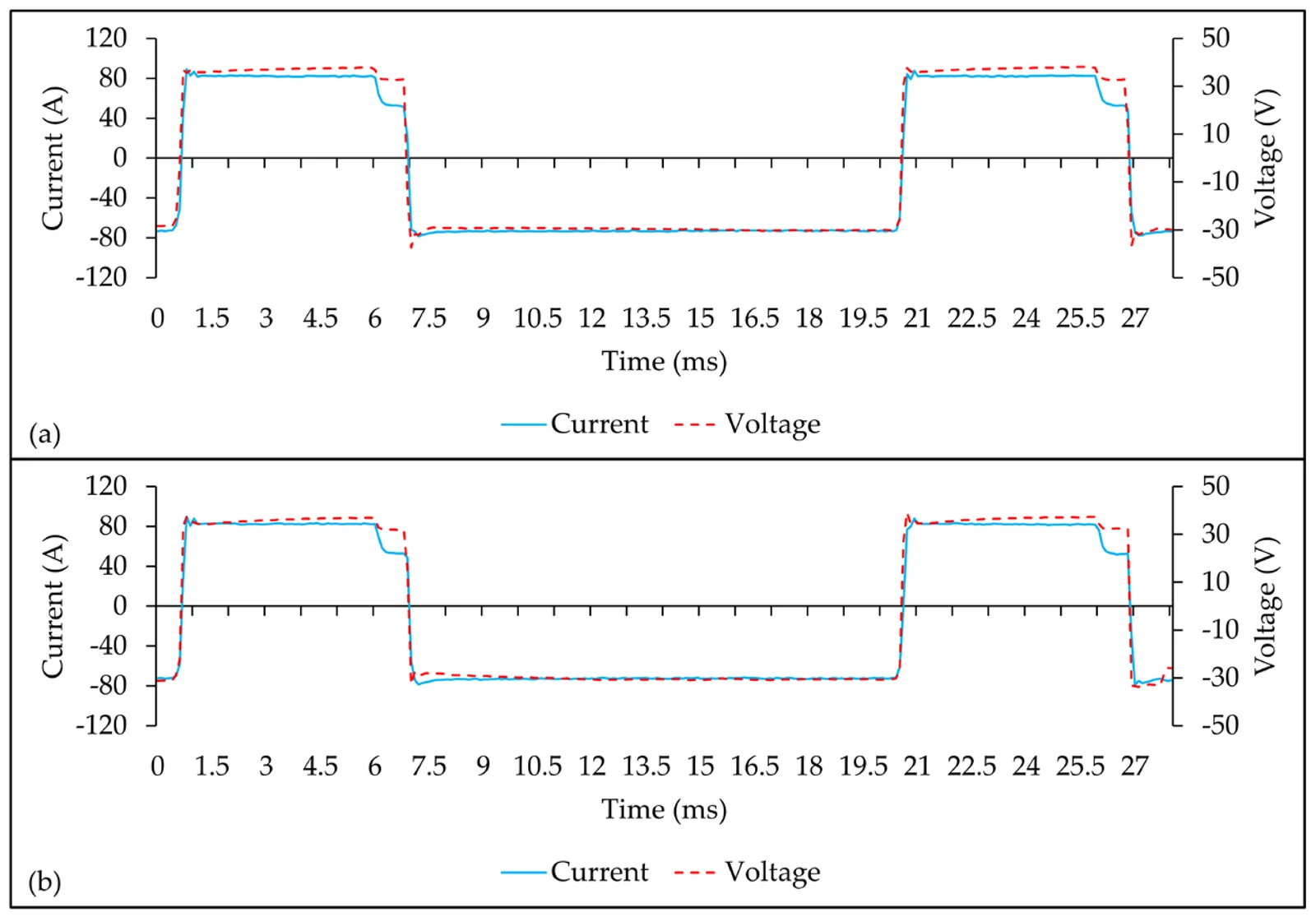
The example of the current and voltage waveforms during the stable electrode negative and electrode positive arcs recorded for the eighth layer at magnetic flux densities of (**a**) 0 mT and (**b**) 3 mT.

Finally, Figure 10 shows the example of the current and voltage waveforms during the stable EN and EP arcs recorded for the eighth layer at MFDs of (a) 0 mT and (b) 3 mT. For both conditions, the preset EN and EP currents were fixed at 80 A and 90 A, respectively.

The current and voltage waveforms exhibited similar profiles under both presented MFD conditions. No significant differences were observed in either the waveform morphology or the average electrical parameters. At 0 mT, the absolute average currents were 73.2 ± 0.8 A and 82.5 ± 1.2 A during the stable EN and EP periods, respectively. The corresponding values at 3 mT were 72.8 ± 0.9 A and 82.8 ± 1.3 A. The absolute average voltages at 0 mT were 29.8 ± 0.8 V and 37.1 ± 0.6 V during the stable EN and EP periods, respectively, whereas those at 3 mT were 30.2 ± 0.9 V and 36.0 ± 0.9 V.

These results indicate that the applied EMF had a negligible influence on the electrical characteristics of the arc. Therefore, the arc deflection induced by EMF did not substantially alter the electrical power supplied by the welding system even though the arc length increased with application of EMF. It implies that total heat input to the wall seems to be almost constant independently of the EMF application. This verification ensures that the obtained reduction in porosity cannot be attributed to changes in the welding electrical characteristics, but rather to the effect of the EMF on CS behavior.

## 4. Conclusions

This study proposed the porosity reduction technology for Wire Arc Additive Manufacturing (WAAM) of aluminum alloys that employs static transversal External Magnetic Field (EMF) to control Cathode Spot (CS) behavior, thereby facilitating bubble release, mainly by accelerating the removal of oxide layer, being a new approach distinct from the conventional methods. In this paper, in particular, effects of EMF strength (Magnetic Flux Density: MFD) on porosity diameter distribution and Porosity Area Percentage (PAP) were discussed. The key findings are summarized as follows:

(1) The pore diameter distributions under different MFDs showed that the number of pores at MFDs of 2 mT and 3 mT decreased to approximately half of that at 0 mT and 1 mT for pore diameter ranging from 20 μm to <60 μm. The results indicate that applying EMF with MFD higher than 2 mT especially contributed to reducing the pore formation in WAAM component.
(2) As MFD increased from 0 mT to 3 mT, PAP decreased from 2.72% to 0.83%, corresponding to an approximately 3.3-fold reduction.
(3) As MFD increased, the electrode negative arc effectively melted the wire, and the electrode positive arc cleaned the oxide layer on rear weld pool surface by CSs.
(4) The High-Speed Video Camera (HSVC) observation also revealed two types of bubble release behavior: a weak eruption outside the arc root (Type A), and a strong eruption inside the arc root (CS) with the appearance of a luminous spot (Type B). Type B is considered to be more effective for porosity reduction than Type A. Type B is thought to be caused by the oxide layer removal by CS. In this case, the gas bubbles trapped beneath the oxide layer could be drawn out strongly through CS, which were frequently observed at 2 mT and 3 mT. This phenomenon was believed to be linked to the significant reduction in porosity at levels of 2 mT and above presented in the results on the pore diameter distributions. This mechanism has been proposed based solely on the results of image analysis, and the evidence is currently insufficient. Therefore, it requires detailed investigation in future research.

The porosity reduction device utilizing EMF proposed in this study is compact and lightweight; because it is designed to be mounted directly onto the WAAM torch, it offers the advantage of easy integration into robotic WAAM systems.

MFD increased up to 3 mT in this study. However, excessively high MFD levels could compromise the stability of the heat source, potentially leading to issues such as insufficient wire melting or agitated weld pool, thereby significantly degrading fabrication accuracy. Since the optimal EMF application conditions change continuously during the process depending on fabrication orientation, process parameters, and the temperature increase during layer deposition, adaptive control of the EMF is expected to enable continuous optimization.

## Figures and Tables

**Table 1:** Chemical composition of the materials used (%mass).

Items	Materials	Type	Mg	Cr	Cu	Fe	Mn	Si	Zn
Substrate	A5052P	Plate	2.20–2.80	0.15–0.35	0.10	0.40	0.10	0.25	0.10
Feedstock material	ER5356	Solid wire	4.50–5.50	0.05–0.20	0.10	0.40	0.05–0.20	0.25	0.10

**Table 2:** Summary of the experimental conditions.

Category	Parameter	Condition	Value
WAAM	Current	Layer 1 (EN/EP)	120 A/110 A
		Layer 2–3 (EN/EP)	100 A/110 A
		Layer 4–6 (EN/EP)	90 A/100 A
		Layer 7–8 (EN/EP)	80 A/90 A
	EP ratio		30%
	Frequency		50 Hz
	Shielding gas	Pure argon	25 L min^−1^
	Plasma gas	Pure argon	1 L min^−1^
	Travel speed		3 mm s^−1^
	Environment temperature		20 °C
	Humidity		~50%
	Wire angle relative to substrate		17.5°
	Wire feed speed	Layer 1	31.8 ± 0.5 mm s^−1^
		Layer 2–3	28.3 ± 0.2 mm s^−1^
		Layer 4–6	28.3 ± 0.2 mm s^−1^
		Layer 7–8	23.7 ± 0.2 mm s^−1^
EMF	Magnetic flux density		0 mT; 1 mT; 2 mT; 3 mT

## Data Availability

The original contributions presented in this study are included in the article. Further inquiries can be directed to the corresponding authors.
